# Photonic Weyl semimetals in pseudochiral metamaterials

**DOI:** 10.1038/s41598-022-23505-1

**Published:** 2022-11-07

**Authors:** Ruey-Lin Chern, Yi-Ju Chou

**Affiliations:** Institute of Applied Mechanics, National Taiwan University, Taipei, 106 Taiwan

**Keywords:** Optical materials and structures, Metamaterials

## Abstract

We investigate the photonic topological phases in pseudochiral metamaterials characterized by the magnetoelectric tensors with symmetric off-diagonal chirality components. The underlying medium is considered a photonic analogue of the type-II Weyl semimetal featured with two pairs of tilted Weyl cones in the frequency-wave vector space. As the ’spin’-degenerate condition is satisfied, the photonic system consists of two hybrid modes that are completely decoupled. By introducing the pseudospin states as the basis for the hybrid modes, the photonic system is described by two subsystems in terms of the spin-orbit Hamiltonians with spin 1, which result in nonzero spin Chern numbers that determine the topological properties. Surface modes at the interface between vacuum and the pseudochiral metamaterial exist in their common gap in the wave vector space, which are analytically formulated by algebraic equations. In particular, the surface modes are tangent to both the vacuum light cone and the Weyl cones, which form two pairs of crossing surface sheets that are symmetric about the transverse axes. At the Weyl frequency, the surface modes that connect the Weyl points form four Fermi arc-like states as line segments. Topological features of the pseudochiral metamaterials are further illustrated with the robust transport of surface modes at an irregular boundary.

## Introduction

Topological phases are new phases of matter characterized by integer quantities known as topological invariants, which remain constants under arbitrary continuous deformations of the system. A good example of the topological phase is the quantum Hall (QH) state^1^, a two-dimensional (2D) electron gas under an external magnetic field, in which the time-reversal (TR) symmetry is broken. A different class of the 2D topological phase in the absence of magnetic field is the quantum spin Hall (QSH) state^2–4^, where the TR symmetry is preserved, and the spin-orbit coupling is responsible for the topological characters. The QH phase is characterized by the Chern number or TKNN invariant^5^, while the QSH phase is characterized by the $Z_2$ invariant^2^ or spin Chern number^6^. At the band gap of a QSH phase, gapless edge states exist for each spin, and the group velocity direction of the edge states is locked by the spin^7^. The spin-momentum locking enables topologically protected edge states that propagate unidirectionally without backscattering^8^. As the edge states are protected by the bulk topology, they are insensitive to small perturbations that do not change the topology. Theoretical concepts developed in the QSH states are generalized to three dimensions (3D), leading to the more general class of 3D topological insulators^9,10^.

In 3D *gapped* topological phases, that is, 3D topological insulators, gapless surface states appear inside the band gap between two topologically distinct bands as in 2D topological phases^11,12^, which can be realized in both TR broken^13,14^ and TR invariant^15–17^ systems. On the other hand, 3D *gapless* topological phases, also known as topological semimetals^18,19^, are new topological phases different from the topological insulators^20–22^, which do not have 2D counterparts. The 3D gapless topological phases are characterized by Weyl degeneracies, which are degeneracies between topologically inequivalent bands. The main signature of 3D gapless topological phases is the appearance of Weyl points, which can exist in systems that lack TR symmetry, inversion symmetry, or both. The Weyl points are understood as the monopoles of Berry curvature in the momentum space that carry quantized topological charges, which are equal to the topological invariants of the system. An important feature of the Weyl points is the existence of Fermi arcs that connect the Weyl points, which correspond to the topologically protected surface states that are robust against disorder. A useful perspective on the Weyl semimetals is to view them as the transitional state between a topological insulator and a trivial insulator^19^.

The novel concepts of topological phases have been extended to photonic systems^23–25^, leading to the discovery of photonic QH states^26–31^, photonic QSH states^32–36^, photonic 3D topological insulators^37–39^, and photonic topological semimetals^40–46^. The key aspect to construct a topological phase is having a Kramers pair in the system, which are doubly degenerate eigenstates under TR symmetry^47^. The Kramers theorem, however, is usually valid for a TR invariant system with spin 1/2^8^ and cannot readily apply to the photonic system with spin 1^48,49^, unless additional symmetry has been imposed. Nevertheless, photons have spin properties as a result of circular polarization^50^. A spin-like quantity called *pseudospin* can be formed by the linear combination of electric and magnetic fields when a specific degenerate condition between the electric and magnetic parameters is satisfied^32^. As a result, the photonic system can be described by an effective Hamiltonian consisting of two subsystems for the pseudospin states^32–34^, and the photonic Kramers pair can be formed in the system. In the presence of chirality or bianisotropy that emulates the spin-orbit coupling, a topological phase can be constructed in the photonic system^51–54^.

In the present study, we investigate the photonic topological phases in *pseudochiral* metamaterials characterized by the magnetoelectric tensors with symmetric off-diagonal chirality components^55–57^. Bulk modes of the underlying medium are represented by two decoupled quadratic equations as a certain symmetry of the material parameters is included. When the ’spin’-degenerate condition^32,34,38^ is satisfied, the bulk modes are featured with two pairs of Weyl cones symmetrically displaced in the frequency-wave vector space. The electromagnetic duality allows for the photonic system to be decoupled as two subsystems for the *hybrid* modes defined as the linear combinations of electric and magnetic fields. By introducing the pseudospin states as the basis for the hybrid modes, the photonic system can be described by a pair of spin-orbit Hamiltonians with spin 1^52–54,58,59^ that respect the fermionic-like *pseudo* time-reversal symmetry. The topological properties of the photonic system are determined by the nonzero spin Chern numbers calculated from the eigenfields of the Hamiltonians. Surface modes at the interface between vacuum and the pseudochiral metamaterial exist in their common gap in the wave vector space, which are analytically formulated by algebraic equations. In particular, the surface modes are tangent to both the vacuum light cone and the Weyl cones, which form two pairs of crossing surface sheets in the frequency-wave vector space. At the Weyl frequency, the surface modes that connect the Weyl points form four Fermi arc-like states as line segments. Finally, the topological features of the pseudochiral metamaterials are illustrated with the robust transport of surface modes at an irregular boundary, which are able to bend around sharp corners without backscattering.

## Results

### Bulk modes

Consider a general bianisotropic medium characterized by the constitutive relations:

1 $$\begin{aligned} {{\mathbf {D}}}&= \varepsilon _0{\underline{\varepsilon }} {{\mathbf {E}}} + \sqrt{{\varepsilon _0}{\mu _0}} {\underline{\xi }} {{\mathbf {H}}}, \end{aligned}$$

2 $$\begin{aligned} {{\mathbf {B}}}&= \mu _0{\underline{\mu }} {{\mathbf {H}}} + \sqrt{{\varepsilon _0}{\mu _0}} {\underline{\zeta }} {{\mathbf {E}}}, \end{aligned}$$

where ${\underline{\varepsilon }}$, ${\underline{\mu }}$, ${\underline{\xi }}$ and ${\underline{\zeta }}$ are frequency-dependent permittivity, permeability, and magnetoelectric tensors, respectively. Treating the combined electric field $\mathbf{E}=(E_x,E_y,E_z)^T$ and magnetic field $\mathbf{H}=(H_x,H_y,H_z)^T$ as six-component vectors, where *T* denotes the transpose, Maxwell’s equations for the time-harmonic electromagnetic fields (with the time convention ${e^{-i\omega t} }$) are written in matrix form as

3 $$\begin{aligned} \left( {\begin{array}{*{20}{l}} {{\omega }{\underline{\varepsilon }}} & {c\mathbf{{k}} \times {\underline{I}} + {{\omega }{\underline{\xi }}}} \\ {-c\mathbf{{k}} \times {\underline{I}} + {{\omega }{\underline{\zeta }}}} & { {\omega }{\underline{\mu }}} \\ \end{array}} \right) \left( {\begin{array}{*{20}{c}} \mathbf{{E}} \\ \mathbf{{H}'} \\ \end{array}} \right) = 0, \end{aligned}$$

where ${\underline{I}}$ is the 3 $\times$ 3 identity matrix, $\mathbf{H}'=\eta _0\mathbf{H}$, with ${\eta _0} = \sqrt{{\mu _0}/{\varepsilon _0}}$. Let the medium be lossless (${{\underline{\varepsilon }}}={{\underline{\varepsilon }}}^\dagger$, ${{\underline{\mu }}}={{\underline{\mu }}}^\dagger$, and ${{\underline{\xi }}}={{\underline{\zeta }}}^\dagger$, where $\dagger$ denotes the Hermitian conjugate) and reciprocal (${{\underline{\varepsilon }}}={{\underline{\varepsilon }}}^T$, ${{\underline{\mu }}}={{\underline{\mu }}}^T$, and ${{\underline{\xi }}}=-{{\underline{\zeta }}}^T$)^55^, which implies that ${{\underline{\varepsilon }}}={{\underline{\varepsilon }}}^*$, ${{\underline{\mu }}}={{\underline{\mu }}}^*$, ${{\underline{\xi }}}=-{{\underline{\xi }}}^*$, and ${{\underline{\zeta }}}=-{{\underline{\zeta }}}^*$, where $*$ denotes the complex conjugate. In the present study, we further assume that the permittivity and permeability tensors are uniaxial: ${\underline{\varepsilon }}=\mathrm{{diag}}\left( {{\varepsilon _t},{\varepsilon _t},{\varepsilon _z}}\right)$, ${\underline{\mu }}=\mathrm{{diag}}\left( {{\mu _t},{\mu _t},{\mu _z}}\right)$, and the magnetoelectric tensors have the following form:

4 $$\begin{aligned} {{\underline{\xi }}} =- {{{\underline{\zeta }}} } = \left( {\begin{array}{*{20}{c}} 0 &{} {i\gamma } &{} 0 \\ { i\gamma } &{} 0 &{} 0 \\ 0 &{} 0 &{} 0 \\ \end{array} } \right) , \end{aligned}$$

where $\varepsilon _n$, $\mu _n$ ($n=t,z$), and $\gamma$ are real-valued quantities. Note that the chirality parameter $\gamma$ appears in the off-diagonal elements of the magnetoelectric tensors ${{\underline{\xi }}}$ and ${{\underline{\zeta }}}$, which means that the magnetoelectric couplings occur in mutually perpendicular directions. The bianisotropic medium characterized by the magnetoelectric tensors with symmetric off-diagonal chirality components as in Eq. (4) is called the *pseudochiral* medium^55^, which has been employed in the study of negative refraction and backward wave^56,57^, and line degeneracy and strong spin-orbit coupling of light^60^ in metamaterials. The underlying medium can be synthesized by two perpendicularly oriented $\Omega$-shape microstructures^55,61,62^ or split ring resonators^60^ embedded in a host medium, or realized with various complex 3D structures^63^. In the pseudochiral medium, the inversion symmetry is broken because of the chirality^44,64^, whereas the TR symmetry is preserved^23^.

The existence of a nontrivial solution of $\mathbf{E}$ and $\mathbf{H}$ requires that the determinant of the 6 $\times$ 6 matrix in Eq. (3) be zero, which gives the characteristic equation of the bulk modes as

5 $$\begin{aligned} {\varepsilon _t}{\mu _t}\left( {k_x^{4} + k_y^{4}} \right) + {\varepsilon _z}{\mu _z}k_z^{4} + 2\left( {{\varepsilon _t}{\mu _t} - 2{\gamma ^2}} \right) k_x^{2} \, k_y^{2} + \left( {{\varepsilon _z}{\mu _t} + {\varepsilon _t}{\mu _z}} \right) (k_x^{2} + k_y^{2})k_z^{2} - \left( {{\varepsilon _t}{\mu _t} - {\gamma ^2}} \right) \left[ {\left( {{\varepsilon _z}{\mu _t} + {\varepsilon _t}{\mu _z}} \right) k_x^{2} + \left( {{\varepsilon _z}{\mu _t} + {\varepsilon _t}{\mu _z}} \right) k_y^2 + 2{\varepsilon _z}{\mu _z}k_z^{2}} \right] k_0^{2} + {\varepsilon _z}{\mu _z}{({\varepsilon _t}{\mu _t} - {\gamma ^2})^2}k_0^{4} = 0, \end{aligned}$$

where ${k_0} = \omega /c$. This is a bi-quadratic equation that incorporates the coupling between transverse electric and transverse magnetic modes. If $\eta _t=\eta _z$, that is, $\sqrt{\mu _t/\varepsilon _t}=\sqrt{\mu _z/\varepsilon _z}$, Eq. (5) can be decoupled as a product of two quadratic equations^60^:

6 $$\begin{aligned} \left( {\frac{{k_x'^2}}{{{a_ + }}} + \frac{{k_y'^2}}{{{a_ - }}} + \frac{{k_z^2}}{b} - k_0^2} \right) \left( {\frac{{k_x'^2}}{{{a_ - }}} + \frac{{k_y'^2}}{{{a_ + }}} + \frac{{k_z^2}}{b} - k_0^2} \right) = 0, \end{aligned}$$

where $k_x'=\left( k_x+k_y\right) /\sqrt{2}$, $k_y'=\left( -k_x+k_y\right) /\sqrt{2}$, ${a_ {\pm } } = \sqrt{{\varepsilon _z}{\mu _z}} \left( {\sqrt{{\varepsilon _t}{\mu _t}} {\pm } \gamma } \right)$, and $b = {\varepsilon _t}{\mu _t} - {\gamma ^2}$. The quadratic equations in Eq. (6) can be of elliptic or hyperbolic type, depending on the sign of the product $a_+ a_-$. There exists a *critical* condition: $|\gamma |=\sqrt{\varepsilon _t\mu _t}$, at which Eq. (5) is simplified to

7 $$\begin{aligned} {\varepsilon _t}{\mu _t}{\left( {k_x^2 - k_y^2} \right) ^2} + \left( {{\varepsilon _t}{\mu _z} + {\varepsilon _z}{\mu _t}} \right) \left( {k_x^2 + k_y^2} \right) k_z^2 + {\varepsilon _z}{\mu _z}k_z^4 = 0, \end{aligned}$$

which is further reduced to two straight lines: $k_x{\pm } k_y=0$ at $k_z=0$. There exist four symmetric points on the two lines: $\left( k_x,k_y\right) =\left( {\pm } \rho ,{\pm } \rho \right)$ and $\left( {\pm } \rho ,{\mp } \rho \right)$, where $\rho = \sqrt{\varepsilon _t\mu _z}k_0$ or $\sqrt{\varepsilon _z\mu _t}k_0$, which serve as the transition points between the elliptic and hyperbolic equations. It will be shown later that these points are identified as the Weyl points in the present problem (cf. “Photonic Weyl system”). In case $\gamma =0$, Eq. (6) is simplified to

8 $$\begin{aligned} {\left( {\frac{{k_x^{\prime 2}+k_y^{\prime 2}}}{{\sqrt{{\varepsilon _t}{\mu _t}{\varepsilon _z}{\mu _z}} }} + \frac{{k_z^{2}}}{{{\varepsilon _t}{\mu _t}}} - k_0^{2}} \right) ^2} = 0, \end{aligned}$$

which is a product of two identical quadratic equations.

Note that the characters of bulk modes may change with the frequency in a dispersive medium (which is usually the case of metamaterials), depending on the choice of frequency range. In the neighborhood of a *reference* frequency $\omega _\text {ref}$, ${\varepsilon _n}$ ($n=t,z$) can be approximated as ${\varepsilon _n} \approx {\varepsilon _{n0}} + {\left. {\frac{{d{\varepsilon _n}}}{{d\omega }}} \right| _{\omega = {\omega _\text {ref}}}}\left( {\omega - {\omega _\text {ref}}} \right) \equiv {\varepsilon _{n0}} + {{{\tilde{\varepsilon }}} _n}\delta \omega /{\omega _\text {ref}}$, where ${{{\tilde{\varepsilon }}} }_n$ is positive definite^58^. A similar relation is valid for $\mu _n$ ($n=t,z$). We further assume that the chirality parameter $\gamma$ varies smoothly around $\omega _{\mathrm{ref}}$ and can be treated as a constant in the analysis^32,52–54^.

### Spin-orbit Hamiltonians

The electromagnetic duality of Maxwell’s equations dictates that the matrix in Eq. (3) holds a symmetric pattern when the ’spin’-degenerate condition: ${\underline{\varepsilon }}={\underline{\mu }}$^32,34,38^ is satisfied. This allows us to rewrite Eq. (3) as

9 $$\begin{aligned} \left( {\begin{array}{*{20}{c}} {{{ {{\mathscr {H}}}_0^+}}} &{} \mathbf{{0}} \\ \mathbf{{0}} &{} {{{ {{\mathscr {H}}}_0^-}}} \\ \end{array}} \right) \left( {\begin{array}{*{20}{c}} {\mathbf{{F}^+} } \\ {\mathbf{{F}^-} } \\ \end{array}} \right) = 0, \end{aligned}$$

where ${{{{\mathscr {H}}}_0^{\pm }}} ={\mp } {\omega }{\underline{\varepsilon }} + i \left( {c\mathbf {k}} \times {\underline{I}}+\omega {\underline{\xi }}\right)$ and $\mathbf{F}^{\pm }={\mathbf{{E}} {\pm } i \mathbf{{H'}}}$ are the *hybrid* modes that linearly combine the electric and magnetic fields. Note that $\mathbf{F}^+$ and $\mathbf{F}^-$ are completely decoupled and determined by two subsystems ($3\times 3$ matrices) with a similar form. By introducing the *pseudospin* states ${\psi _ {\pm } } = {U^{ - 1}}{{{\tilde{\psi }}} _ {\pm } }$ as the basis for the hybrid modes, where $\tilde{\psi _ {\pm } } = {\left( - {\frac{{ {F_x^{\pm }} {\mp } i{F_y^{\pm }}}}{{\sqrt{2} }},{F_z},\frac{{{F_x^{\pm }} {\pm } i{F_y^{\pm }}}}{{\sqrt{2} }}} \right) ^T}$ and $U = \mathrm{{diag}}\left( {\sqrt{{{{{\tilde{\varepsilon }}} }_z}/{{{{\tilde{\varepsilon }}} }_t}} ,1,\sqrt{{{{{\tilde{\varepsilon }}} }_z}/{{{{\tilde{\varepsilon }}} }_t}} } \right)$, Eq. (9) can be formulated as a pair of eigensystems when the frequency dispersion of the medium near the reference frequency $\omega _\text {ref}$ is taken into account. In the isotropic case, where ${\varepsilon _{t0}} = {\varepsilon _{z0}} \equiv \varepsilon$ and ${{{\tilde{\varepsilon }}} _t} = {{{\tilde{\varepsilon }}} _z} \equiv {{\tilde{\varepsilon }}}$, the eigensystems for Eq. (9) are given by (see “Spin-orbit Hamiltonians”)

10 $$\begin{aligned} {\mathscr {H}_ {\pm } }{\psi _ {\pm } } - {{\mathscr {D}}}_{\pm }{\psi _ {\pm } } = {\pm }\delta \omega {\psi _ {\pm } }, \end{aligned}$$

where

11 $$\begin{aligned} {{\mathscr {H}}_ + } = v \mathbf{{k}}\cdot \mathbf{{S}},\quad {{\mathscr {H}}_ - } = - v \mathbf{{k}}\cdot \mathbf{{S}}^*, \end{aligned}$$

and ${{\mathscr {D}} }_{\pm } = {\pm }{\omega _{\mathrm{ref}}} \left( {\varepsilon {\underline{I}}-\gamma \{S_x,S_y\}}\right) /{{\tilde{\varepsilon }}}$. Here, $v=c/{{{{\tilde{\varepsilon }}} }}$, $\mathbf{{k}}=k_x{\hat{x}}+k_y{\hat{y}}+k_z{\hat{z}}$, $\mathbf{{S}} = {S_x}{\hat{x}} + {S_y}{\hat{y}} + {S_z}{\hat{z}}$, $S_n$ ($n=x,y,z$) are the spin matrices for spin 1, and $\{A,B\}=AB+BA$ is the anticommutator. Note that Eq. (10) is formulated as an eigensystem with $\delta \omega$ being the eigenvalue. The Hamiltonian ${{\mathscr {H}}}_{\pm }$ in Eq. (11) represents the spin-orbit coupling $\mathbf{{k}}\cdot \mathbf{{S}}$ with spin 1, which is mathematically equivalent to the Hamiltonian of a magnetic dipole moment in the magnetic field^58^.

### Topological invariants

The topological properties of the spin-orbit Hamiltonians ${{\mathscr {H}}}_{\pm }$ can be characterized by the topological invariants using the eigenfields. For this purpose, we calculate the Berry flux over a closed surface in the wave vector space. The eigensystem for the Hamiltonian ${{\mathscr {H}}}_{\pm }$ in Eq. (11):

12 $$\begin{aligned} {\mathscr {H}_ {\pm } }\psi _ {\pm } ^\sigma =\lambda _ {\pm } ^\sigma \psi _ {\pm } ^\sigma \end{aligned}$$

is solved to give the eigenvalues $\lambda _ {\pm } ^\sigma$ and eigenvectors $\psi _ {\pm } ^\sigma$ ($\sigma ={\pm } 1, 0$), based on which the Chern numbers are calculated to give (see “Topological invariants”)

13 $$\begin{aligned} {C_{\sigma }} = 2 \sigma . \end{aligned}$$

The nonzero $C_\sigma$ ($\sigma ={\pm } 1$) characterize the topological properties of the system, where $\sigma$ refers to the helicity (or handedness) of the pseudospin states. In particular, the surface or edge states at the interface between two distinct topological phases are topologically protected, which means that their existence is guaranteed by the difference in band topology on two sides of the interface. In this system, the total Chern number $C=\sum \limits _\sigma {{C_\sigma }}=0$ and the spin Chern number $C_{\mathrm{spin}}=\sum \limits _\sigma {{\sigma C_\sigma }}=4$, which are consistent with the quantum spin Hall effect of light^50^. The spin Chern number indicates that there exist two pairs of QSH edge states which are doubly-degenerate with respect to the helicity $\sigma$. The existence of surface modes in Maxwell’s equations, however, requires the presence of an interface (between two different media) that breaks the duality symmetry of electromagnetic fields as in an unbounded region, and therefore only one pair of edge modes survives at the interface^50,59^. The topological invariants remain unchanged under arbitrary continuous deformations of the system. The topological properties in the isotropic case will be retained when a certain anisotropy is included in the system. For a more general anisotropic case, the exact calculation of topological invariants can be obtained by the numerical integration of Berry curvatures^65^.

### Pseudo time-reversal symmetry

The Hamiltonian for Maxwell’s equations [cf. Eq. (3)] in the pseudochiral medium, which is lossless and reciprocal, is TR invariant under $T_b$, that is,

14 $$\begin{aligned} \left( {T_b \otimes {\underline{I}}}\right) {{{\mathscr {H}}}_m \left( \mathbf{k} \right) }\left( {T_b \otimes {\underline{I}}}\right) ^{ - 1} = {{{\mathscr {H}}}_m\left( -\mathbf{k} \right) }, \end{aligned}$$

where

15 $$\begin{aligned} {{{\mathscr {H}}}_m \left( \mathbf{k} \right) } = \left( {\begin{array}{*{20}{l}} {{\omega }{\underline{\varepsilon }}} & {c\mathbf{{k}} \times {\underline{I}} + {{\omega }{\underline{\xi }}}} \\ {-c\mathbf{{k}} \times {\underline{I}} + {{\omega }{\underline{\zeta }}}} & { {\omega }{\underline{\mu }}} \\ \end{array}} \right) , \end{aligned}$$

${T_b} = {\sigma _z}K$ (with $T_b^2=1$) is the bosonic TR operator for photons^23^, with *K* being the complex conjugation, and $\otimes$ denotes the tensor product. The Hamiltonian ${{\mathscr {H}}}_m$, however, is not TR invariant under $T_f$, that is, $\left( {T_f \otimes I}\right) {{{\mathscr {H}}}_m \left( \mathbf{k} \right) }\left( {T_f \otimes I}\right) ^{ - 1} \ne {{{{\mathscr {H}}}}_m\left( -\mathbf{k} \right) }$, where ${T_f} = {i\sigma _y}K$ (with $T_f^2=-1$) is the fermionic TR operator for electrons^23^. Nevertheless, the combined Hamiltonian formed by two spin-orbit Hamiltonians ${{\mathscr {H}}}_{\pm }$ [cf. Eq. (11)] is TR invariant under $T_p$, that is,

16 $$\begin{aligned} \left( {T_p \otimes {\underline{I}}}\right) {{{{\mathscr {H}}}}_c \left( \mathbf{k} \right) }\left( {T_p \otimes {\underline{I}}}\right) ^{ - 1} = {{{{\mathscr {H}}}}_c \left( -\mathbf{k} \right) }, \end{aligned}$$

where

17 $$\begin{aligned} {{{\mathscr {H}}}_c}\left( {{\mathbf {k}}} \right) = \left( {\begin{array}{*{20}{c}} {v {{{\mathbf {k}}}\cdot {{\mathbf {S}}}} } &{} {{\mathbf {0}}} \\ {{\mathbf {0}}} &{} { - v {{ {{{\mathbf {k}}}\cdot {{\mathbf {S}}}} }^*}} \\ \end{array} } \right) , \end{aligned}$$

and ${T_p}$ is the fermionic-like *pseudo* TR operator having the same form of $T_f$. The pseudo TR operator $T_p$ is inspired by noticing that $\mathbf{{E}} \leftrightarrow \mathbf{{H}}$ during the TR operation, which is defined as ${T_p} = {T_b}{\sigma _x} = {\sigma _z}K{\sigma _x} = i{\sigma _y}K$ with $T_p^2=-1$^34^. Here, $\sigma _x=\left( 0,1;1,0\right)$, $\sigma _y=\left( 0,-i;i,0\right)$, and $\sigma _z=\mathrm{diag}\left( 1,-1\right)$ are the Pauli matrices. The pseudo TR symmetry of the combined Hamiltonian ${{{\mathscr {H}}}}_c$ is crucial in determining the topological phases in the photonic system of spin 1, which allows for the existence of bidirectional propagating spin-polarized edge states as in electronic systems.

### Surface modes

Let the *xy* plane be an interface between vacuum ($z>0$) and the pseudochiral metamaterial ($z<0$) characterized by $\varepsilon _n=\varepsilon$, $\mu _n=\mu$ ($n=t,z$), and ${\pm }\gamma$ (cf. “Bulk modes”), at which the surface modes may exist. According to Maxwell’s boundary conditions: the continuity of tangential electric and magnetic field components at the interface, the characteristic equation of surface modes can be analytically formulated by using the eigenfields of bulk modes on two sides of the interface, which is given as (see “Surface wave equation”)

18 $$\begin{aligned} & \varepsilon \mu k_0^2\left( {2\varepsilon \mu k_x^2 - 2\left( {{\gamma ^2} - 2} \right) k_y^2- 2k_z^{(1)}k_z^{(2)} + \left( {\varepsilon + \mu } \right) k_z^{(0)}\left( {k_z^{(1)} + k_z^{(2)}} \right) } \right) \\ {}&- k_y^2\left( {2k_x^2 + 2k_y^2 - 2\varepsilon \mu k_z^{(1)}k_z^{(2)} + \left( {\varepsilon + \mu } \right) k_z^{(0)}\left( {k_z^{(1)} + k_z^{(2)}} \right) } \right) \\&- 2\varepsilon \mu \left( {\varepsilon \mu - {\gamma ^2}} \right) k_0^4 - 2i\gamma \varepsilon \mu k_0^3\left( {\left( {\varepsilon + \mu } \right) k_z^{(0)} - k_z^{(1)} - k_z^{(2)}} \right) \\ {}&+ 2i{k_0}{k_y}\left[ \sqrt{\varepsilon \mu } \left( {\varepsilon \mu - 1} \right) {k_x}\left( {k_z^{(1)} - k_z^{(2)}} \right) + \gamma {k_y}\left( {\left( {\varepsilon + \mu } \right) k_z^{(0)} - \varepsilon \mu \left( {k_z^{(1)} + k_z^{(2)}} \right) } \right) \right] = 0, \end{aligned}$$

where $k_z^{(0)} = \sqrt{k_0^2 - k_x^2 - k_y^2}$ is the normal wave vector component (to the interface) in vacuum,$k_z^{(1)} = - \sqrt{\left( {\varepsilon \mu - {\gamma ^2}} \right) k_0^2 - k_x^2 + \frac{{2\gamma }}{{\sqrt{\varepsilon \mu } }}{k_x}{k_y} - k_y^2}$ and $k_z^{(2)} = - \sqrt{\left( {\varepsilon \mu - {\gamma ^2}} \right) k_0^2 - k_x^2 - \frac{{2\gamma }}{{\sqrt{\varepsilon \mu } }}{k_x}{k_y} - k_y^2}$ are the normal wave vector components in the pseudochiral metamaterial, and the superscripts (1) and (2) refer to two independent polarizations.

**Figure 1:**
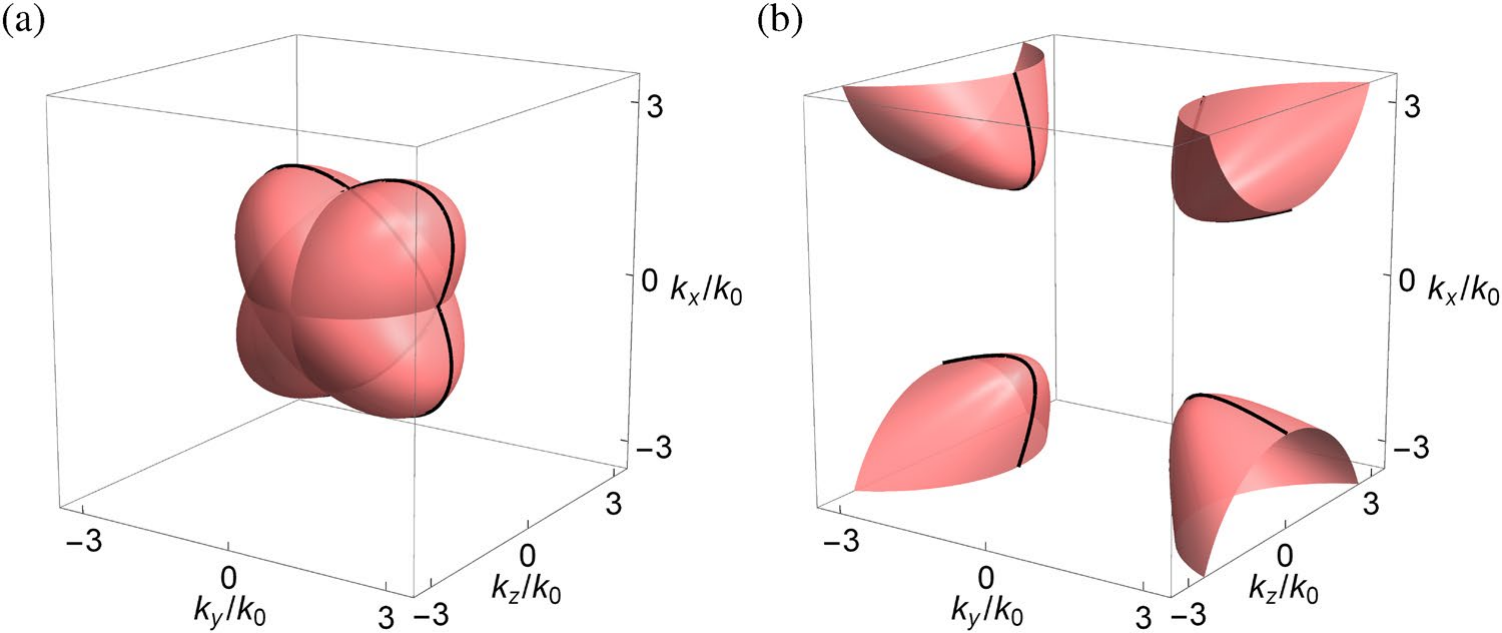
Equifrequency surfaces of bulk modes in the wave vector space for the pseudochiral metamaterial with (**a**) $\varepsilon _n=\mu _n=2$ and $\gamma ={\pm } 1$ (**b**) $\varepsilon _n=\mu _n=1.5$ and $\gamma ={\pm } 2$ ($n=t,z$). Black contours are bulk modes at $k_z=0$ (cf. Fig. 2).

## Discussion

### Bulk modes

Figure 1 shows the equifrequency surfaces of bulk modes in the wave vector space for the pseudochiral metamaterial based on Eq. (6). Regarding the relative magnitude of chirality parameter (to the geometric mean of permittivity and permeability), the bulk modes can be classified into two phases:

(I) For $|\gamma |<\sqrt{\varepsilon \mu }$, the bulk modes are represented by two intersecting ellipsoids in the wave vector space, as shown in Fig. 1a. The major and minor axes of the two ellipsoids are mutually perpendicular in the *xy* plane, which make an angle of ${\pm } 45^\circ$ with respect to the $k_x$ or $k_y$ axis.
(II) For $|\gamma |>\sqrt{\varepsilon \mu }$, the bulk modes are represented by a pair of two-sheeted hyperboloids in the wave vector space, as shown in Fig. 1b. The major and minor axes of the hyperboloids coincide with those of the ellipsoids in phase (I). There exists a gap between the bulk modes, which is the region enclosed by four vertices of the hyperboloids in the $k_x$–$k_y$ plane: $\left( {{k_x},{k_y}} \right) = \left( { {\pm } \rho {k_0}, {\pm } \rho {k_0}} \right)$ and $\left( { {\pm } \rho {k_0}, {\mp } \rho {k_0}} \right)$, where $\rho = \sqrt{\left( {\varepsilon \mu + \gamma \sqrt{\varepsilon \mu } } \right) /2}$.

Recall that the effective Hamiltonian in the present problem consists of two subsystems of the hybrid modes. Each subsystem is described by the spin-orbit Hamiltonian with spin 1 (cf. “Spin-orbit Hamiltonians”) and characterized by nonzero topological invariants (cf. “Topological invariants”). In this regard, the pseudochiral metamaterial is considered a photonic analogue of the topological phase.

### Surface modes

**Figure 2:**
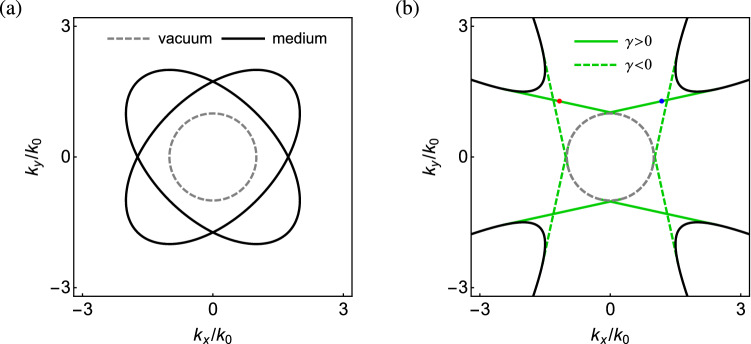
Surface modes at the interface between vacuum and the pseudochiral metamaterial with (**a**) $\varepsilon _n=\mu _n=2$ and $\gamma ={\pm } 1$ (**b**) $\varepsilon _n=\mu _n=1.5$ and $\gamma ={\pm } 2$ ($n=t,z$). Gray dashed contour is dispersion circle of vacuum. Black contours are bulk modes at $k_z=0$ (cf. Fig. 1). In (**b**), blue and red dots are chosen points for surface wave simulations (cf. Fig. 4).

Figure 2 shows the surface modes at the interface between vacuum ($z>0$) and the pseudochiral metamaterial ($z<0$) in the $k_x$–$k_y$ plane based on Eq. (18). The bulk modes at $k_z=0$ (cf. Fig. 1) are also shown in the plots. Regarding the relative magnitude of chirality parameter (to the geometric mean of permittivity and permeability), there are two situations for the surface modes to be addressed:

(i) For $|\gamma |<\sqrt{\varepsilon \mu }$, where the bulk modes are in phase (I), the surface modes do not exist, for there is no common gap between vacuum and the pseudochiral metamaterial, as shown in Fig. 2a. At $k_z=0$, the bulk modes are represented by two intersecting ellipses, while the vacuum dispersion is represented by a circle.
(ii) For $|\gamma |>\sqrt{\varepsilon \mu }$, where the bulk modes are in phase (II), the surface modes are represented by two pairs of crossing line segments, with the reflection symmetry about the $k_x$ ($k_y$) axis for $\gamma >0$ ($\gamma <0$), as shown in Fig. 2b. Note that the crossing point for each pair of line segments is close to the vacuum dispersion circle. The surface modes for $\gamma >0$ (green solid lines) and $\gamma <0$ (green dashed lines) are further symmetric with respect to the major or minor axes of the hyperbolas (the bulk modes at $k_z=0$). These axes are in fact the bulk modes at the critical condition $|\gamma |=\sqrt{\varepsilon \mu }$ (cf. “Bulk modes”), which are represented by two straight lines: $k_x{\pm } k_y=0$ at $k_z=0$ plane [cf. Eq. (7)].

In the present configuration, the bulk modes for opposite sign of the chirality parameter are identical because of the symmetry about $\gamma$ [cf. Eqs. (5) or (6)]. The surface modes are located in the common gap of the bulk modes in the wave vector space, that is, outside the bulk modes of vacuum and the pseudochiral metamaterial. All the surface modes are *tangent* to the bulk modes^19,20^, including the the vacuum dispersion circle: $k_x^2+k_y^2=k_0^2$ [cf. gray dashed contours in Fig. 2b] and the pseudochiral metamaterial [cf. black solid contours in Fig. 2b]. This feature follows from the fact that the surface modes must convert seamlessly into the bulk modes as they approach their termination points^66^. The evanescent depth of the surface mode grows until at the point where the surface mode merges with the bulk mode^19^. The bulk modes on the vacuum side are topologically trivial, while on the pseudochiral medium side they are topologically nontrivial with nonzero topological invariants (cf. “Topological invariants”). The surface modes correspond to the topological phase transition between two distinct topological phases in the momentum space^52,67^, their existence being guaranteed by the bulk-edge correspondence. In particular, the Hamiltonian of the photonic system respects the pseudo TR symmetry (cf. “Pseudo time-reversal symmetry”), leading to the topological protection of photonic surface or edge states.

### Photonic Weyl system

Let the frequency dependence of the pseudochiral medium be characterized by the Lorentz dispersion models: $\varepsilon = \varepsilon _\infty - \omega _p^2/\left( \omega ^2 - \omega _0^2 \right)$ and $\mu = \mu _\infty - \Omega _\mu \omega ^2/\left( \omega ^2-\omega _0^2\right)$, which are usually employed in the study of metamaterials^68^. Here, $\omega _0$ is the the resonance frequency and $\omega _p$ is the effective plasma frequency of the medium. The chirality parameter is given by $\gamma = \Omega _\gamma \omega \omega _{p}/\left( \omega ^2 - \omega _0^2 \right)$, where $\Omega _\gamma ^2=\Omega _\mu$^69,70^. This model guarantees that the energy density in the underlying medium is positive definite (see “Electromagnetic energy density”).

**Figure 3:**
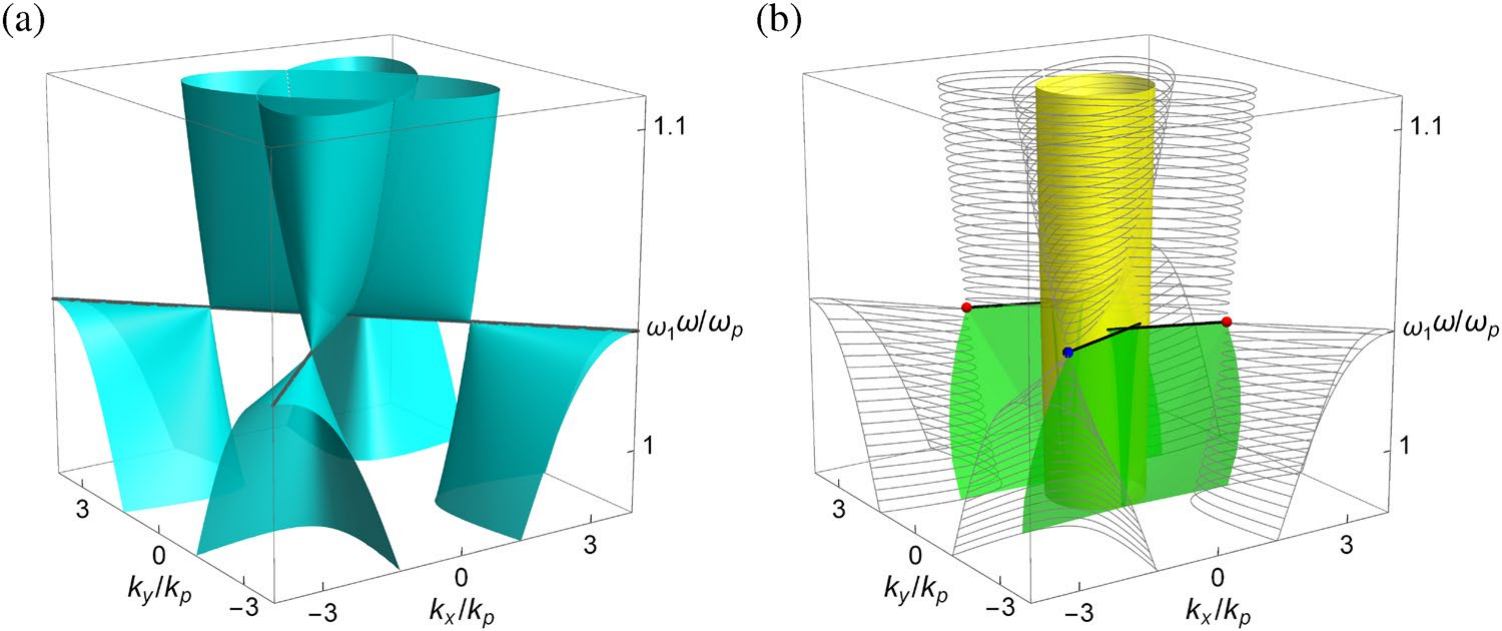
Dispersion of (**a**) bulk modes and (**b**) surface modes in the frequency-wave vector space for the pseudochiral metamaterial with $\varepsilon _{\infty }=4$, $\mu _{\infty }=3$, $\Omega _\mu =0.522$, $\Omega _\gamma ={\pm }0.723$ and $\omega _0/\omega _p=0.8$. Wave vector components are scaled by $k_p=\omega _p/c$. In (**a**), dark gray lines are bulk modes at the Weyl frequency. In (**b**), bulk modes at constant frequencies are outlined in gray mesh. Yellow cylinder is the dispersion surface of vacuum. Blue and red dots are the Weyl points with opposite chirality. Black lines are the Fermi arcs.

Figure 3a shows the dispersion of bulk modes for the pseudochiral metamaterial in the frequency-wave vector space with $k_z=0$. The bulk modes consist of two pairs of tilted conic surfaces symmetrically displaced in the $k_x$–$k_y$ plane. Each branch of the conic surface contains an elliptic surface in phase (I) and a hyperbolic surface in phase (II) (cf. “Bulk modes”). In the present configuration, the material parameters are arranged such that $\varepsilon =\mu =\gamma =\frac{{{\varepsilon _\infty }{\mu _\infty }}}{{{\varepsilon _\infty } + {\mu _\infty }}}$ at the frequency $\omega _1= \sqrt{\omega _0^2 + \left( {{\varepsilon _\infty } + {\mu _\infty }} \right) \omega _p^2/\varepsilon _\infty ^2}$, where the bulk modes are reduced to two straight lines: $k_x{\pm } k_y=0$ at $k_z=0$ [cf. Eq. (7)]. This is the condition that fulfills both the ‘spin’-degenerate condition (cf. “Spin-orbit Hamiltonians”) and the critical condition (cf. “Bulk modes”) in the present medium, which also forms the point-like degeneracy in the bulk modes. Here, $\omega _1$ is the transition frequency between phase (I) and phase (II), at which $|\gamma |=\sqrt{\varepsilon \mu }$. For $\omega >\omega _1$, the bulk modes are composed of ellipses, while for $\omega <\omega _1$, the bulk modes are composed of hyperbolas. The former and the latter touch at four *saddle* points: $\left( {{k_x},{k_y}} \right) = \left( {\pm } {\rho _1}, {\pm } {\rho _1} \right)$ and $\left( {\pm } \rho _1, {\mp } {\rho _1} \right)$, where ${\rho _1} = \frac{{{\mu _\infty }}}{{c\left( {{\varepsilon _\infty } + {\mu _\infty }} \right) }}\sqrt{\varepsilon _\infty ^2\omega _0^2 + \left( {{\varepsilon _\infty } + {\mu _\infty }} \right) \omega _p^2}$. In this situation, the dispersion of bulk modes resembles the linear crossing of valence and conduction bands in the *Weyl semimetal*^71^, with the crossing points known as the *Weyl points* and the associated frequency $\omega _1$ as the *Weyl frequency*. In the present configuration, the Weyl points all exist at the same frequency^72^, which are known as the *ideal* Weyl points^44,46,73–76^. At the Weyl frequency, the bulk modes are reduced to straight lines, which are similar to the boundaries between electrons and hole pockets^19^. In this regard, the underling medium is considered a photonic analogue of the type-II Weyl semimetal^77^.

Note that in the absence of chirality ($\gamma =0$), the bulk modes are featured with the Dirac cone with fourfold degeneracy at the Dirac point: $(k_x,k_y,\omega )=(0,0,\omega _1)$ at the center of the wave vector space [cf. Eq. (8)]. The present medium in the situation, however, is not a Dirac semimetal since this degeneracy is not topologically protected^19^. In the presence of chirality ($\gamma \ne 0$), the inversion symmetry is broken (cf. “Bulk modes”) and the fourfold degeneracy is lifted. As a result, the bulk modes are featured with two pairs of Weyl cones with twofold degeneracy at four Weyl points [cf. Eq. (6)]. For a TR symmetric system, the total number of Weyl points must be a multiple of four^19^, since under the time reversal a Weyl point at $\mathbf{k}_0$ is converted to a Weyl point at $-\mathbf{k}_0$ with the same chirality. As the net chirality should vanish, there must be another pair of Weyl points with the opposite chirality. A similar feature of four Weyl points has also been observed in electronic^78^ and photonic^44^ systems. The topological charges carried by the Weyl points are consistent with the nonzero topological invariants $C_{\pm }={\pm } 2$ in the present system (cf. “Topological invariants”). Here, the topological charges ${\pm } 2$ are associated with the *unconventional* spin-1 Weyl points with threefold linear degeneracy^46,79–81^. The net chirality vanishes in the Weyl semimetal, which agrees with the fact that the total Chern number is zero (cf. “Topological invariants”).

Figure 3b shows the dispersion of surface modes at the interface between vacuum and the pseudochiral metamaterial in the frequency-wave vector space. For comparison, the bulk modes (with $k_z=0$) at constant frequencies are outlined in gray mesh. Different from the surface modes in topological insulators that exist in the frequency (energy) band gap, the surface modes in gapless topological semimetals are defined in the region free of bulk modes at the same frequency (energy)^19^. Recall that the surface modes exist only in phase (II), where $|\gamma |>\sqrt{\varepsilon \mu }$ (cf. “Surface modes”). The surface modes are therefore located below $\omega _1$, at which $|\gamma |=\sqrt{\varepsilon \mu }$. Because of the frequency dependence of material parameters, the dispersion of surface modes is shown to be bended. The surface modes form two pairs of bended surface sheets tangent to both and the Weyl cones and the vacuum light cone in the frequency-wave vector space. At the Weyl frequency, the edge states that connect the Weyl points form the so-called *Fermi arcs*. In the present configuration, two pairs of Fermi arc-like states are represented by four line segments, each of which connects the Weyl point on one end and the vacuum dispersion surface on the other [cf. black line in Fig. 3b]. A similar feature of two pairs of Fermi arcs has also been observed in the photonic Dirac semimetal^68,82^.

**Figure 4:**
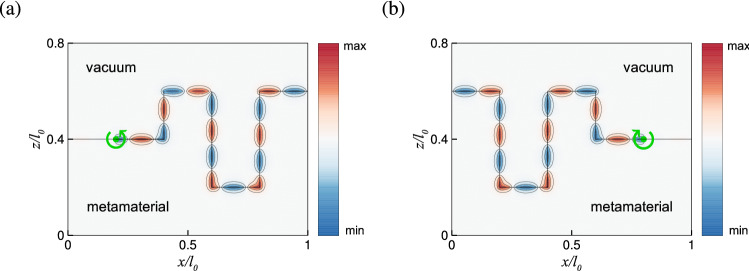
Surface wave propagation at the interface between vacuum and the pseudochiral metamaterial with $\varepsilon =\mu =1.5$, $\gamma =2$, and $k_y/k_0=1.28$ for (**a**) right-handed and (**b**) left-handed circular polarization, which correspond to the red and blue dots, respectively, in Fig. 2b. Green dot is the position of dipole source. Circular arrow denotes the handedness of circular polarization. Red and blue colors correspond to positive and negative values of Re[$E_y$], respectively, and *x* and *z* coordinates are scaled by $l_0\approx 12.43\lambda _0$, with $\lambda _0=2\pi /k_0$.

Finally, the topological features of the pseudochiral metamaterial are illustrated with the propagation of surface waves at an irregular boundary^51–54,59,65,83,84^. For this purpose, a dipole source is placed at the interface between vacuum and the pseudochiral metamaterial to excite the surface modes in the their common band gap (outside the bulk modes in the wave vector space), so that the waves are evanescent away from the interface on both sides. In Fig. 4, a pair of surface modes are excited at $k_y/k_0=1.28$ [cf. blue and red dots in Fig. 2(b)] with right- or left-handed circular polarizations (see “Simulation”), which correspond to the opposite helicity of topological edge states. The surface waves propagate unidirectionally to the right or left along an irregular boundary with sharp corners. In particular, the surface waves counterpropagate at the boundary for different handednesses of circular polarization. This feature is consistent with the characteristic of surface modes in the present configuration [cf. Fig. 2b], in which there exist a positive $k_z$ (blue dot) and a negative $k_z$ (red dot) for a fixed $k_x$. The surface waves are able to bend around sharp corners without backscattering, which demonstrates that the edge states are topologically protected.

In conclusion, we have investigated the photonic topological phases in pseudochiral metamaterials characterized by the magnetoelectric tensors with symmetric off-diagonal chirality components. The photonic system is described by a pair of spin-orbit Hamiltonians with spin 1 in terms of the pseudospin states, and the topological properties are determined by the nonzero spin Chern numbers. Surface modes exist at the interface between vacuum and the pseudochiral metamaterial, which depict the typical features of topological edge states between two distinct topological phases. The underlying medium is regarded as a photonic analogue of the type-II Weyl semimetal featured with two pairs of Weyl cones and the associated Fermi arc-like states. Topological features of the pseudochiral metamaterials are illustrated with the robust transport of surface modes at an irregular boundary.

## Methods

### Spin-orbit Hamiltonians

The wave equation for the hybrid modes $\mathbf{F^{\pm }}={\mathbf{{E}}{\pm } i \mathbf{{H'}}}$ in Eq. (9) can be rewritten as

19 $$\begin{aligned} {\tilde{{\mathscr {H}}_ {\pm }} }{{{\tilde{\psi }}} _ {\pm } } = {\tilde{{{\mathscr {D}}}}_ {\pm } }{\tilde{\psi _ {\pm }} }, \end{aligned}$$

where

20 $$\begin{aligned} {\tilde{{\mathscr {H}}_ {\pm }} } = {\pm } c\left( {\begin{array}{*{20}{c}} {{k_z}} &{} {\frac{{{k_x} {\mp } i{k_y}}}{{\sqrt{2} }}} &{} 0 \\ {\frac{{{k_x} {\pm } i{k_y}}}{{\sqrt{2} }}} &{} 0 &{} {\frac{{{k_x} {\mp } i{k_y}}}{{\sqrt{2} }}} \\ 0 &{} {\frac{{{k_x} {\pm } i{k_y}}}{{\sqrt{2} }}} &{} { - {k_z}} \end{array} } \right) ,\quad {\tilde{{{\mathscr {D}}}}_{\pm } } = {\pm }\omega \left( {\begin{array}{*{20}{c}} {{\varepsilon _t}} &{} 0 &{} {i\gamma } \\ 0 &{} {{\varepsilon _z}} &{} 0 \\ { - i\gamma } &{} 0 &{} {{\varepsilon _t}} \\ \end{array}} \right) , \end{aligned}$$

and $\tilde{\psi _ {\pm } } = {\left( - {\frac{{ {F_x^{\pm }} {\mp } i{F_y^{\pm }}}}{{\sqrt{2} }},{F_z},\frac{{{F_x^{\pm }} {\pm } i{F_y^{\pm }}}}{{\sqrt{2} }}} \right) ^T}$ are the *pseudospin* states that include ${\pm }\pi /2$ phase difference between the transverse hybrid field components (with respect to the optical axis of the medium)^58^. In the neighborhood of a *reference* frequency $\omega _\text {ref}$, ${\varepsilon _n}$ ($n=t,z$) can be approximated as ${\varepsilon _n} \approx {\varepsilon _{n0}} + {\left. {\frac{{d{\varepsilon _n}}}{{d\omega }}} \right| _{\omega = {\omega _\text {ref}}}}\left( {\omega - {\omega _\text {ref}}} \right) \equiv {\varepsilon _{n0}} + {{{\tilde{\varepsilon }}} _n}\delta \omega /{\omega _\text {ref}}$, where ${{{\tilde{\varepsilon }}} }_n$ is positive definite^58^. Taking into account of the frequency dispersion of the medium near the reference frequency, Eq. (19) is rearranged as a pair of eigensystems:

21 $$\begin{aligned} {{\mathscr {H}}'_ {\pm } }{\psi _ {\pm } } - {{{{\mathscr {D}}}}'_{\pm } }{\psi _ {\pm } } = {\pm }\delta \omega {\psi _ {\pm } }, \end{aligned}$$

where

22 $$\begin{aligned} {{{{\mathscr {H}}}}_ {\pm }' } = {\pm }\frac{c}{{\sqrt{{{{{\tilde{\varepsilon }}} }_t}{{{{\tilde{\varepsilon }}} }_z}} }}\left( {\begin{array}{*{20}{c}} {\sqrt{\frac{{{{{{\tilde{\varepsilon }}} }_z}}}{{{{{{\tilde{\varepsilon }}} }_t}}}} }{k_z} &{} {\frac{{ {k_x} {\mp } i{k_y}}}{{\sqrt{2} }}} &{} 0 \\ {\frac{{ {k_x} {\pm } i{k_y}}}{{\sqrt{2} }}} &{} 0 &{} {\frac{{ {k_x} {\mp } i{k_y}}}{{\sqrt{2} }}} \\ 0 &{} {\frac{{ {k_x} {\pm } i{k_y}}}{{\sqrt{2} }}} &{} - {\sqrt{\frac{{{{{{\tilde{\varepsilon }}} }_z}}}{{{{{{\tilde{\varepsilon }}} }_t}}}} }{k_z} \\ \end{array} } \right) ,\quad {{{{\mathscr {D}}}}'_{\pm } } ={\pm } {\omega _{\mathrm{{ref}}}}\left( {\begin{array}{*{20}{c}} {\frac{{{\varepsilon _{t0}}}}{{{{{{\tilde{\varepsilon }}} }_t}}}} &{} 0 &{} {\frac{{i\gamma }}{{{{{{\tilde{\varepsilon }}} }_t}}}} \\ 0 &{} {\frac{{{\varepsilon _{z0}}}}{{{{\tilde{\varepsilon }}} _z}}} &{} 0 \\ -{\frac{{i\gamma }}{{{{{{\tilde{\varepsilon }}} }_t}}}} &{} 0 &{} {\frac{{{\varepsilon _{t0}}}}{{{{{{\tilde{\varepsilon }}} }_t}}}} \\ \end{array}} \right) , \end{aligned}$$

and ${\psi _ {\pm } } = {U^{ - 1}}{{{\tilde{\psi }}} _ {\pm } }$ with $U = \mathrm{{diag}}\left( {\sqrt{{{{{\tilde{\varepsilon }}} }_z}/{{{{\tilde{\varepsilon }}} }_t}} ,1,\sqrt{{{{{\tilde{\varepsilon }}} }_z}/{{{{\tilde{\varepsilon }}} }_t}} } \right)$. In the isotropic case, where ${\varepsilon _{t0}} = {\varepsilon _{z0}} \equiv \varepsilon$ and ${{{\tilde{\varepsilon }}} _t} = {{{\tilde{\varepsilon }}} _z} \equiv {{\tilde{\varepsilon }}}$, Eq. (21) is simplified to

23 $$\begin{aligned} {{\mathscr {H}}_ {\pm } }{\psi _ {\pm } } - {{{\mathscr {D}}}}_{\pm }{\psi _ {\pm } } = {\pm }\delta \omega {\psi _ {\pm } }, \end{aligned}$$

where

24 $$\begin{aligned} {{{\mathscr {H}}}}_ + = v {\mathbf{{k}}\cdot \mathbf{{S}},\quad {{{\mathscr {H}}}}_ - = - v \mathbf{{k}}\cdot \mathbf{{S}} ^*}, \end{aligned}$$

and $${{{\mathscr {D}}} }_{\pm } = {\pm }{\omega _{\mathrm{ref}}} \left( {\varepsilon {\underline{I}}-\gamma \{S_x,S_y\}}\right) /{{\tilde{\varepsilon }}}$$. Here, $$v=c/{{{{\tilde{\varepsilon }}} }}$$, $$\mathbf{k}=k_x{\hat{x}}+k_y{\hat{y}}+k_z{\hat{z}}$$, $$\mathbf{{S}} = {S_x}{\hat{x}} + {S_y}{\hat{y}} + {S_z}{\hat{z}}$$,

25 $$\begin{aligned} {S_x} = \frac{1}{{\sqrt{2} }}\left( {\begin{array}{*{20}{c}} 0 &{} 1 &{} 0 \\ 1 &{} 0 &{} 1 \\ 0 &{} 1 &{} 0 \\ \end{array}} \right) ,\quad {S_y} = \frac{1}{{\sqrt{2} }}\left( {\begin{array}{*{20}{c}} 0 &{} { - i} &{} 0 \\ i &{} 0 &{} { - i} \\ 0 &{} i &{} 0 \\ \end{array}} \right) ,\quad {S_z} = \left( {\begin{array}{*{20}{c}} 1 &{} 0 &{} 0 \\ 0 &{} 0 &{} 0 \\ 0 &{} 0 &{} { - 1} \\ \end{array}} \right) \end{aligned}$$

are the spin matrices for spin 1, and $\{A,B\}=AB+BA$ is the anticommutator.

### Topological invariants

The Hamiltonian ${{{\mathscr {H}}}}_{\pm }$ [cf. Eq. (24)] on the sphere *S*: $|\mathbf{k}|=k_0$ is rewritten in spherical coordinates as

26 $$\begin{aligned} {{{{\mathscr {H}}}}_ {\pm } } = {\pm } \frac{{v {k_0}}}{{\sqrt{2} }}\left( {\begin{array}{*{20}{c}} {\sqrt{2} \cos \theta } &{} {{e^{ {\mp } i\phi }}\sin \theta } &{} 0 \\ {{e^{ {\pm } i\phi }}\sin \theta } &{} 0 &{} {{e^{ {\mp } i\phi }}\sin \theta } \\ 0 &{} {{e^{ {\pm } i\phi }}\sin \theta } &{} { - \sqrt{2} \cos \theta } \\ \end{array}} \right) , \end{aligned}$$

where $\theta$ and $\phi$ are the polar and azimuthal angles, respectively. The eigensystem for the Hamiltonian ${{{\mathscr {H}}}}_{\pm }$:

27 $$\begin{aligned} {{\mathscr {H}}_ {\pm } }\psi _ {\pm } ^\sigma =\lambda _ {\pm } ^\sigma \psi _ {\pm } ^\sigma \end{aligned}$$

is solved to give the eigenvalues $\lambda _ {\pm } ^\sigma = \sigma v k_0$ ($\sigma ={\pm } 1, 0$) and the normalized eigenvectors as

28 $$\begin{aligned}&\psi _ {\pm } ^\sigma = \frac{1}{2}\left( {\begin{array}{*{20}{c}} {{\pm }\sigma {e^{ {\mp } 2i\phi }}\left( {{\pm }\sigma + \cos \theta } \right) } \\ {{\pm }\sigma \sqrt{2} {e^{ {\mp } i\phi }}\sin \theta } \\ { 1 {\mp } \sigma \cos \theta } \\ \end{array} } \right) \quad (\sigma ={\pm } 1), \end{aligned}$$

29 $$\begin{aligned}&\psi _ {\pm } ^\sigma = \left( {\begin{array}{*{20}{c}} { - {e^{ {\mp } 2i\phi }}\sin \theta } \\ {\sqrt{2} {e^{ {\mp } i\phi }}\cos \theta } \\ {\sin \theta } \\ \end{array} } \right) \quad (\sigma =0). \end{aligned}$$

Based on Eqs. (28) and (29), the Berry connections $\mathbf{A}_{\pm }^\sigma =-i\left\langle {{\psi _{\pm } ^ \sigma }} \right. \left| {\nabla {\psi _{\pm } ^ \sigma }} \right\rangle$ are obtained as

30 $$\begin{aligned}&\mathbf{A}_{\pm }^\sigma ={\mp }\frac{1}{r}\left( \cot \frac{\theta }{2}\right) ^{{\pm } \sigma }{{\hat{\phi }}}\quad (\sigma ={\pm } 1), \end{aligned}$$

31 $$\begin{aligned}{\mathbf{A}}_{\pm }^{\sigma} ={\mp }\frac{1}{r}\csc \theta {{\hat{\phi }}}\quad (\sigma =0). \end{aligned}$$

The Berry curvatures $\mathbf{F}_\sigma =\nabla \times \mathbf{A}_{\pm }^\sigma$ are then given by

32 $$\begin{aligned} \mathbf{F}_\sigma =\sigma \frac{{{\hat{r}}}}{{{r^2}}}\quad (\sigma ={\pm } 1, 0). \end{aligned}$$

Integrating over the unit sphere *S*, the Chern numbers ${C_\sigma } = \frac{1}{2\pi }\int _S {\mathbf{F}_\sigma \cdot d\mathbf{{s}}}$ are calculated to give

33 $$\begin{aligned} {C_\sigma } = 2\sigma \quad (\sigma ={\pm } 1, 0). \end{aligned}$$

### Surface wave equation

According to Maxwell’s equations, the eigenfields on either side of the interface ($z=0$) are given by the nontrivial solutions of $\mathbf{E}$ and $\mathbf{H}$ [cf. Eq. (3)] or the *null space* of ${{{\mathscr {H}}}}_m$ [cf. Eq. (15)]. On the vacuum side ($z>0$), we have

34 $$\begin{aligned}&{\mathbf{{E}}^{(1)}} = \frac{1}{{k_0^2}}\left( { - {k_x}{k_y},k_0^2 - k_y^2, - {k_y}k_z^{(0)}} \right) ,\quad {\mathbf{{H}}^{(1)}} = \frac{1}{{{\eta _0}{k_0}}}\left( { - k_z^{(0)},0,{k_x}} \right) , \end{aligned}$$

35 $$\begin{aligned}&{\mathbf{{E}}^{(2)}} = \frac{1}{{k_0^2}}\left( {{k_x}k_z^{(0)},{k_y}k_z^{(0)}, - k_x^2 - k_y^2} \right) ,\quad {\mathbf{{H}}^{(2)}} = \frac{1}{{{\eta _0}{k_0}}}\left( { - {k_y},{k_x},0} \right) , \end{aligned}$$

where $k_z^{(0)} = \sqrt{k_0^2 - k_x^2-k_y^2}$ is the normal wave vector component (to the interface) in vacuum. On the pseudochiral medium side ($z<0$), the eigenfields are given by

36 $$\begin{aligned}&{\mathbf{{E}}^{(3)}} = \frac{1}{{k_0^2}}\left( { - \sqrt{\varepsilon }\mu \left( {{\beta _ - }{k_0} - i{k_x}{k_z^{(1)}}} \right) ,\frac{{\sqrt{\mu }{\delta _ - }\left( {\varepsilon \mu k_0^2 - k_y^2} \right) }}{{{\alpha _ - }{k_0} - i{k_y}{k_z^{(1)}}}}, - i\sqrt{\mu }{\delta _ - }} \right) , \end{aligned}$$

37 $$\begin{aligned}&{\mathbf{{H}}^{(3)}} = \frac{1}{{{\eta _0}k_0^2}}\left( {\varepsilon \sqrt{\mu }\left( { - i{\beta _ - }{k_0} - {k_x}{k_z^{(1)}}} \right) ,\frac{{i\sqrt{\varepsilon }{\delta _ - }\left( {\varepsilon \mu k_0^2 - k_y^2} \right) }}{{{\alpha _ - }{k_0} - i{k_y}{k_z^{(1)}}}},\sqrt{\varepsilon }{\delta _ - }} \right) , \end{aligned}$$

38 $$\begin{aligned}&{\mathbf{{E}}^{(4)}} = \frac{1}{{k_0^2}}\left( { - \sqrt{\varepsilon }\mu \left( {{\beta _ + }{k_0} + i{k_x}{k_z^{(2)}}} \right) ,\frac{{\sqrt{\mu }{\delta _ + }\left( {\varepsilon \mu k_0^2 - k_y^2} \right) }}{{{\alpha _ + }{k_0} + i{k_y}{k_z^{(2)}}}},i\sqrt{\mu }{\delta _ + }} \right) , \end{aligned}$$

39 $$\begin{aligned}&{\mathbf{{H}}^{(4)}} = \frac{1}{{{\eta _0}k_0^2}}\left( {\varepsilon \sqrt{\mu }\left( {i{\beta _ + }{k_0} - {k_x}{k_z^{(2)}}} \right) , - \frac{{i\sqrt{\varepsilon }{\delta _ + }\left( {\varepsilon \mu k_0^2 - k_y^2} \right) }}{{{\alpha _ + }{k_0} + i{k_y}{k_z^{(2)}}}},\sqrt{\varepsilon }{\delta _ + }} \right) , \end{aligned}$$

where $${\alpha _ {\pm } } = \sqrt{\varepsilon \mu } {k_x} {\pm } \gamma {k_y}$$, $${\beta _ {\pm } } = \sqrt{\varepsilon \mu } {k_y} {\pm } \gamma {k_x}$$, $${\delta _ {\pm } } = \sqrt{\varepsilon \mu } k_x^2 {\pm } 2\gamma {k_x}{k_y} + \sqrt{\varepsilon \mu } k_y^2$$, $$k_z^{(1)} = - \sqrt{\left( {\varepsilon \mu - {\gamma ^2}} \right) k_0^2 - k_x^2 + \frac{{2\gamma }}{{\sqrt{\varepsilon \mu } }}{k_x}{k_y} - k_y^2}$$ and $$k_z^{(2)} = - \sqrt{\left( {\varepsilon \mu - {\gamma ^2}} \right) k_0^2 - k_x^2 - \frac{{2\gamma }}{{\sqrt{\varepsilon \mu } }}{k_x}{k_y} - k_y^2}$$ are the normal wave vector components in the pseudochiral medium, and the superscripts (1) and (2) refer to two independent polarizations. Note that the eigenfields in Eqs. (34)–(39) share the common tangential wave vector components $$k_x$$ and $$k_y$$ across the interface, as a direct consequence of the phase matching of electromagnetic fields. For the surface waves to exist on the vacuum side ($$z>0)$$, $$k_z^{(0)}$$ should be purely imaginary with a positive value, so that the waves decay exponentially away from the interface. On the pseudochiral medium side ($$z<0$$), $$k_z^{(1)}$$ and $$k_z^{(2)}$$ should be purely imaginary with a negative value for a similar reason.

The tangential electric and magnetic field components are continuous at the interface:

40 $$\begin{aligned}&C_1E_n^{(1)} + C_2E_n^{(2)} = C_3E_n^{(3)} + C_4E_n^{(4)}, \end{aligned}$$

41 $$\begin{aligned}&C_1H_n^{(1)} + C_2H_n^{(2)} = C_3H_n^{(3)} + C_4H_n^{(4)}, \end{aligned}$$

where $n=x,y$ and $C_1$, $C_2$, $C_3$, $C_4$ are constants. The existence of a nontrivial solution of these constants requires that the determinant of the 4 $\times$ 4 matrix obtained from Eqs. (40) and (41) be zero, which gives the characteristic equation of the surface mode as

42 $$\begin{aligned} &\varepsilon \mu k_0^2\left( {2\varepsilon \mu k_x^2 - 2\left( {{\gamma ^2} - 2} \right) k_y^2 - 2k_z^{(1)}k_z^{(2)} + \left( {\varepsilon + \mu } \right) k_z^{(0)}\left( {k_z^{(1)} + k_z^{(2)}} \right) } \right) \\&- k_y^2\left( {2k_x^2 + 2k_y^2 - 2\varepsilon \mu k_z^{(1)}k_z^{(2)} + \left( {\varepsilon + \mu } \right) k_z^{(0)}\left( {k_z^{(1)} + k_z^{(2)}} \right) } \right) \\&- 2\varepsilon \mu \left( {\varepsilon \mu - {\gamma ^2}} \right) k_0^4 - 2i\gamma \varepsilon \mu k_0^3\left( {\left( {\varepsilon + \mu } \right) k_z^{(0)} - k_z^{(1)} - k_z^{(2)}} \right) \\&+ 2i{k_0}{k_y}\left[ {\sqrt{\varepsilon \mu } \left( {\varepsilon \mu - 1} \right) {k_x}\left( {k_z^{(1)} - k_z^{(2)}} \right) + \gamma {k_y}\left( {\left( {\varepsilon + \mu } \right) k_z^{(0)} - \varepsilon \mu \left( {k_z^{(1)} + k_z^{(2)}} \right) } \right) } \right] = 0. \end{aligned}$$

### Electromagnetic energy density

The time-averaged energy density in a lossless medium is given by^85^

43 $$\begin{aligned} \left\langle W \right\rangle =\frac{ 1}{4}{V^\dag }{M}V, \end{aligned}$$

where

44 $$\begin{aligned} {M} = \left( {\begin{array}{*{20}{c}} {\frac{{\partial (\omega {\varepsilon })}}{{\partial \omega }}} &{} 0 &{} 0 &{} 0 &{} {i\frac{{\partial (\omega {\gamma })}}{{\partial \omega }}} &{} 0 \\ 0 &{} {\frac{{\partial (\omega {\varepsilon })}}{{\partial \omega }}} &{} 0 &{} {i\frac{{\partial (\omega {\gamma })}}{{\partial \omega }}} &{} 0 &{} 0 \\ 0 &{} 0 &{} {\frac{{\partial (\omega {\varepsilon })}}{{\partial \omega }}} &{} 0 &{} 0 &{} 0 \\ 0 &{} {-i\frac{{\partial (\omega {\gamma })}}{{\partial \omega }}} &{} 0 &{} {\frac{{\partial (\omega {\mu })}}{{\partial \omega }}} &{} 0 &{} 0 \\ { - i\frac{{\partial (\omega {\gamma })}}{{\partial \omega }}} &{} 0 &{} 0 &{} 0 &{} {\frac{{\partial (\omega {\mu })}}{{\partial \omega }}} &{} 0 \\ 0 &{} 0 &{} 0 &{} 0 &{} 0 &{} {\frac{{\partial (\omega {\mu })}}{{\partial \omega }}} \\ \end{array}} \right) \end{aligned}$$

and $V=\left( \varepsilon _0 E_x,\varepsilon _0 E_y,\varepsilon _0 E_z,\mu _0 H_x,\mu _0 H_y,\mu _0 H_z \right) ^T$, with ${V^\dag }$ being the Hermitian conjugate of *V*. The energy density must be positive definite, which implies that both the trace and the determinant of *M* are positive:

45 $$\begin{aligned} \mathrm{Tr}\left( {M}\right)>0,\quad \mathrm{Det}\left( {M}\right) >0. \end{aligned}$$

Based on the Lorentz dispersion models used in the present medium (cf. “Photonic Weyl system”), these quantities become

46 $$\begin{aligned} {{\mathrm{Tr}}}\left( {{M}} \right) =\frac{3}{{{{\left( {{\omega ^2} - \omega _0^2} \right) }^2}}}\left[ \left( {\varepsilon _{\infty }} + {\mu _{\infty }} \right) {{\left( {{\omega ^2} - \omega _0^2} \right) }^2} + \left( {{\omega ^2} + \omega _0^2} \right) \omega _p^2 - \omega \left( {{\omega ^2} - 3\omega _0^2} \right) {{\Omega _{\mu }}}\right] \end{aligned}$$

and

47 $$\begin{aligned} &\mathrm{{Det}}\left( {{M}} \right) = \frac{1}{{{{\left( {{\omega ^2} - \omega _0^2} \right) }^{12}}}} \cdot \\&\left( {{\varepsilon _{\infty }}\left( {{\omega ^2} - \omega _0^2} \right) + \left( {{\omega ^2} + \omega _0^2} \right) \omega _p^2} \right) \left( {{\mu _{\infty }}{{\left( {{\omega ^2} - \omega _0^2} \right) }^2} - {\omega ^2}\left( {{\omega ^2} - 3\omega _0^2} \right) {\Omega _{\mu }}} \right) \cdot \\&{\left[ {\left( {{\varepsilon _{\infty }}{{\left( {{\omega ^2} - \omega _0^2} \right) }^2} + \left( {{\omega ^2} + \omega _0^2} \right) \omega _p^2} \right) \left( {{\mu _{\infty }}{{\left( {{\omega ^2} - \omega _0^2} \right) }^2} - {\omega ^2}\left( {{\omega ^2} - 3\omega _0^2} \right) {\Omega _{\mu }}} \right) - 4{\omega ^2}\omega _0^4\omega _p^2\Omega _\gamma ^2} \right] ^2}, \end{aligned}$$

both of which are positive in the present study.

### Simulation

Let the *xz* plane be the simulation domain with $k_y$ being the out-of-plane wave vector component, which is kept fixed in the simulation so that the eigenwaves possess the same $k_y$^51^. In this manner, the simulation domain is considered a section plane (normal to the interface) of the 3D space. The surface wave is excited at a certain point on the boundary between vacuum and the pseudochiral medium, which can be implemented experimentally by a dipole antenna^27,86^. For the dipole to serve as the source of circularly polarized waves, two in-plane components with ${\pm }\pi /2$ phase difference are included to excite the right-handed or left-handed wave^87^.

## Data Availability

No datasets were generated or analysed during the current study.
